# Assessment of Sex-Related Behaviours, Human Immunodeficiency Virus (HIV) Knowledge and Sexually Transmitted Infections (STIs) among Men of Reproductive Age in Cameroon

**DOI:** 10.3390/ijerph111212726

**Published:** 2014-12-09

**Authors:** Abayomi Samuel Oyekale

**Affiliations:** Department of Agricultural Economics and Extension, North-West University Mafikeng Campus, Mmabatho 2735, South Africa; E-Mail: asoyekale@gmail.com

**Keywords:** sex-related behaviours, reproductive age, Human Immunodeficiency Virus (HIV) knowledge, Cameroon

## Abstract

Sexually transmitted infections (STIs), including human immunodeficiency virus (HIV), are among the major public health challenges in Cameroon. This paper determined the effect of men’s sex-related behaviors and HIV knowledge on reported STIs. The data came from the 2012 Cameroon’s Demographic and Health Survey (DHS) that were collected from 7191 respondents in 2012. Descriptive and logistic regression methods were used for data analysis. Results showed that majority of the respondents were aware of STIs and Acquired Immune Deficiency Syndrome (AIDS), while 3.96% reported STIs. Also, 49.45% of the men had no wife, while 75.58% and 84.58% noted that condoms and keeping of one partner could be used to prevent HIV transmission, respectively. Wrong impressions that mosquito bites and sharing of food could lead to HIV infection were held by 31.94% and 12.44% of the men, respectively. Among those that reported STIs, 33.33%, 30.18% and 13.33% respectively used condom during sex with most recent partner, second to most recent partner and third to most recent partner, compared to 24.69%, 15.04% and 4.17% among those that did not report STIs. Logistic regression results showed that probability of STI increased significantly (*p* < 0.05) with condom use with third most recent partners, being married, wrong knowledge that mosquito bites cause HIV and being away for more than one month, while it significantly reduced (*p* < 0.05) with number of children, knowledge that having one partner prevents STIs. It was concluded that policy initiatives and programmes to enhance right sexual knowledge and behavior among men would go a long way in reducing STI incidence in Cameroon.

## 1. Introduction

In Cameroon, sexually transmitted infections (STIs), including human immunodeficiency virus (HIV), are issues at the forefront of public health challenges [1,2]. With a high HIV prevalence rate (5.1% in 2010), Cameroon records one of the highest burdens of associated socio-economic impacts of HIV in sub-Saharan Africa (SSA) [3]. The onus therefore rests on policy makers to provide adequate mechanisms for addressing the forces behind the high vulnerability of the population to the risk of HIV and other sexually transmitted infections (STIs) [2,3]. However, not much research has been previously carried out in this area. 

Although known to have afflicted the human race for many centuries, the discovery of HIV in the early 1980s shone substantial light on the epidemiology of several venereal diseases broadly classified as sexually transmitted infections (STIs). This was essentially motivated by the positive association between patients’ clinical diagnoses with STIs and HIV status [4,5]. It had also been proposed that the presence of some STI pathogens provides quick transmission routes for HIV. However, despite many recent developments in the health delivery systems which are geared towards understanding their epidemiology, STIs are still among most notable global public health challenges [6]. This is directly linked to the inability to fully address the socio-behavioural issues that motivate infection [5]. 

Caused by more than 30 pathogens [5], STIs transcend most other known infectious diseases in both frequency and severity. Their major mode of transmission which is unprotected sexual intercourse subjects mankind to higher vulnerability due to perpetually exhibited weaknesses in sex-related issues. Despite the cognitive notion of their deserved privacy, it is definitely difficult to give STIs the required secrecy because of their diversely inflicted pains [7]. Given their associated morbidity and mortality, and with or without inclusion of HIV&AIDS, the ultimate impact of STIs on global economic growth and development cannot be overemphasized [5,6,7]. The World Health Organization (WHO) estimated that globally, about 340 million new STI infections are reported annually [8]. STIs (HIV inclusive) also result in loss of more than 51 million years of healthy life globally [9]. In developing countries, however, STIs account for 17% of total economic losses that can be traced to ill-health [10].

Several conceptual frameworks had been used by health professionals to curtail the spread of STIs. The dynamic transmission model is notable among these approaches [7]. This model emphasizes rapid changes in individual’s sex-related behaviours through drastic reduction in the number of sexual partners, desire to use condoms during intercourse and timeliness in seeking treatment from authentic healthcare providers [7]. For several reasons, persons infected with STIs often seek some confidentiality in their treatment-seeking behavior. Where this cannot be appropriately guaranteed, infection dynamics increases, with serious socio-economic consequences. In addition, the operational model of STI control highlights the different processes involved before an infection can be considered to be perfectly cured. This model noted that inability to prevent risky sexual behavior, lack of access to health facilities, inability to properly identify and treat infected persons and deficiency on the part of healthcare system to provide the right treatments are critical in explaining the persistent rise in STIs. It also pinpoints compliance with prescribed treatment as a guarantee that diagnosed infection would be properly treated [7]. 

Specifically, sexuality and associated behaviours are the principal motivators of STIs. Due to inexperience and likelihood of involvement in risky sexual acts, adolescents often exhibit higher vulnerability to STIs [11]. This matter is often made worse because non-acceptability of the consequences of their sexual behaviours to health workers, colleagues and their parents makes it difficult for many of these adolescents to seek timely and effective treatment for STIs. There is also gender disparity in infection frequency between adolescent males and females with the latter being more vulnerable. This is usually the case because more often, adolescent females would date men of older ages who are more likely to infect them with STIs contracted from other sex partners [12].A study in Yaoundé reported that between 15%–25% of adolescent boys in the year 2000 and 14%–18% in 2002 were infected by STIs [13]. In addition, it was found that girl children bore higher burden of STIs with average of 46%–55% in year 2000 and 34%–36% in 2002 [13].

It should be further emphasized that several cultural issues are critical motivating factors for STI transmission. Precisely, the influence of cultural norms on what is sexually acceptable within the context of societal development has a strong influence on human sexuality by putting some form of barriers on some risky sexual behaviours [14]. Therefore, failure of several cultural norms to properly promote less risky sexual behavior is a major antecedent of sexual promiscuousness and transmission of STIs [15,16]. Obviously, if cultural norms are not able to adequately address extension of age sexual debut, there is higher tendency of STI risk [17,18].

Although persistent upsurge in socio-economic impacts of HIV and AIDS has necessitated reduction in involvement in risky sexual behaviours, individuals can also exhibit some carefree attitudes that subject them to serious vulnerability [17,18]. Among married couples, the omen of marital infidelity is often associated with domestic violence and divorce. Similarly, within marriage, some men are prone to contract STIs from their wives who may have contracted it through multiple sexual partners or inadequate sanitary condition of shared toilets. Therefore, in the absence of marital faithfulness or engagement of a man with many legally recognized marital partners in a polygamous marriage relationship, STI transmission rate can be significantly higher. Furthermore, polygamy as a factor for STI transmission becomes significant if some of the wives engage in extra-marital activities [19]. In this case, the rate of infection is high and partner notification (PN) which is a compulsory screening and treatment of all partners of infected person becomes significantly difficult due to high tendency of seeking confidentiality in matters related to STIs’ diagnosis and treatments [20,21].

Furthermore, due to several physiological changes that accompany pregnancy, pregnant women possess higher risk of being infected with STIs [20]. Therefore, husbands of such women may be susceptible to higher STIs due to the lower likelihood of condom use. It may also be argued that pregnant women with husbands that have multiple sexual partners may exhibit higher vulnerability due to low probability of using condoms [21]. Literature also documents the relevance of habits like drinking and smoking. Precisely, involvement in periodic or regular drinking of alcohol exposes the person to higher likelihood of having unprotected sex.

This paper therefore analyzed the effect of involvement in risky sexual activities and HIV knowledge on reported STIs among men of reproductive age in Cameroon. This will add to existing literature and provide veritable platform for policy making on some necessary social and public health interventions. It was hypothesized that involvement in risky sexual behavior and possession of adequate HIV knowledge do not significantly reduce STIs. In the remainder of the paper, the materials and methods, results of data analyses, discussions of major findings, and recommendations are presented.

## 2. Materials and Methods

### 2.1. Data and Sampling Methods

The Cameroon’s Demographic and Health Survey (DHS) data for 2011 were used in this study. The data are representative of Cameroon as a whole judging from the coverage and sampling procedures. Two-stage stratified sampling method was used to select the samples with sample size proportionally related to estimated population. Data were collected from all the 10 administrative regions while respondents from Yaounde and Douala were separated from Central and Lithoral administrative regions, respectively. At the first stage of sampling, 580 primary Enumeration Areas (EAs) were selected following the list from the third General Census of Population and Housing Census (PHC) in 2005. The breakdown of selected EAs showed that 289 were from rural areas, while 291 were from urban. At the second stage of sampling, households that belonged to each EA constituted the sampling units. In total, 578 EAs were sampled comprising of 14,214 households. Within these households, 7191 men of reproductive ages between 15 and 49 years were interviewed [22]. The data were properly weighted in order to ensure representativeness.

### 2.2. Model Specification

In order to model the correlates of reported STIs in the previous one year as probed by the questionnaire, logistic regression model was used. This was warranted by the dichotomous nature of the responses which were coded 1 if yes and 0 otherwise. The estimated model can be presented as [23]:

(1) $$logit\;\left(P\right)=\text{ln}\left(\frac{P}{1-P}\right)$$

(2) $$\frac{P}{1-P}=e^{\text{α}+\text{β}X}$$

(3) $$P=\frac{e^{\text{α}+\text{β}X}}{1+e^{\text{α}+\text{β}X}}$$

The dependent variable *P* (coded as yes = 1, 0 otherwise) represents probability of reporting STI. In the estimation of the model, *α* and *β* are the estimated parameters. The independent variables (*X_i_*) were screened for multicollinearity and those finally included are urban residence (yes = 1, 0 otherwise), years of education, gender of household head (male =1, 0 otherwise), age of household head, read newspapers (yes = 1, 0 otherwise), listen to radio (yes = 1, 0 otherwise), watch television (yes = 1, 0 otherwise), number of times away from home in the past one year, away from home for more than one month in the past one year (yes = 1, 0 otherwise), partner pregnant (yes = 1, 0 otherwise), number of living children, knowledge of any contraceptive method (yes = 1, 0 otherwise), Smoke nothing (yes = 1, 0 otherwise), circumcised (yes = 1, 0 otherwise), married (yes = 1, 0 otherwise), use condom to prevent HIV (yes = 1, 0 otherwise), have one sexual partner to prevent HIV (yes = 1, 0 otherwise), mosquito bites give HIV (yes = 1, 0 otherwise), sharing of food gives HIV (yes = 1, 0 otherwise), healthy person can have HIV (yes = 1, 0 otherwise), condom use with second most recent sexual partner (yes = 1, 0 otherwise), condom use with third most recent sexual partner (yes = 1, 0 otherwise) and number of sexual partners excluding spouse in the last one year.

## 3. Results

Table 1 presents the demographic characteristics of the men that were interviewed. It reveals that 3.96% was infected with STIs in the one year before the survey. In the combined data, the average age was 30.74 years. The average age of the men that reported STIs was 31.37 years, which can be compared with 30.71 years computed for those without infection. In the combined data, about 50.42% of the men lived in urban areas. Also, 65.25% of the men that reported STIs were married.

**Table 1:** Demographic characteristics and sexual behaviour of respondents.

Variables	No STI (*n* = 6906)		Reported STI (*n* = 285)		Total (*n*= 7191)	
	Mean	Std Dev	Mean	Std Dev	Mean	Std Dev
Sex of household head	87.13	--	84.91	--	87.04	--
Age of household head	44.35	14.232	42.53	14.679	44.28	14.253
Current age of respondent	30.71	12.217	31.37	10.153	30.74	12.142
Residence in urban areas	50.29	--	53.68	--	50.42	--
Number of wives/partners	0.59	0.679	0.61	0.610	0.59	0.676
Current marital status	55.29	0.497	65.25	0.477	55.68	0.497
Currently residing with wife/partner	41.59	0.493	43.51	0.497	41.66	0.493
Total number of years of education	7.56	4.920	8.33	3.794	7.59	4.883
Number of household members	6.59	4.440	5.96	4.271	6.56	4.435
Reading newspaper or magazine	46.09	0.499	54.39	0.499	46.42	0.499
Listening to radio	80.47	0.396	83.16	0.375	80.57	0.396
Frequency of watching television	82.84	0.377	90.18	0.298	83.13	0.374
Times away from home in last 12 months	5.48	12.406	7.95	14.099	5.57	12.486
Away for more than one month in last 12 months	20.06	0.400	28.77	0.453	20.44	0.403
Partner currently pregnant	7.85	0.269	8.42	0.278	7.87	0.269
Number of living children	2.24	3.102	2.01	2.546	2.23	3.082
Knowledge of any contraceptive method	97.87	0.144	99.65	0.059	97.94	0.142
Smokes nothing	83.26	0.373	75.09	0.433	82.94	0.376
Respondent circumcised	95.26	0.212	98.60	0.118	95.40	0.210
Number of sexual partners excluding spouse in the past twelve months	0.76	2.124	2.14	3.762	0.82	2.228

While the average number of wives was 0.59 for the combined data, men that reported STIs had slightly higher average number of wives (0.61). Average year of education for all the men was 7.59, although infected men had slightly higher average years of education (8.33). Among men that reported STI infections, average household size was 5.96, while that for non-infected ones was 6.59. Male-headed households constituted 84.91% among those that reported infections, as against 87.13% for non-infected. In the combined data, the average age of household heads was 44.28 years, while that for men with STIs infection it was 42.53 years. 

Among men that reported STIs, 54.39%, 83.16% and 90.18% read newspapers, listened to news, and watched television, respectively, as against 46.09%, 80.47% and 82.84% for those that did not report infection. Average time away from home in the past one year for those with STI infection was 7.95, while 29.00% were away for more than one month in the previous one year. In the combined data and among uninfected men, 7.87% of the wives were pregnant, while majority of the respondents (infected or not) had knowledge of contraceptives. Non-smoking habit was reported by 75.09% of the men that reported STIs as against 83.26% by those that did not report. Also, 98.60% of the respondents that reported STIs were circumcised, as against 95.26% in the combined data. 

Table 2 indicates that 49.45% of all respondents had no wife. Among the men that reported STIs and those that did not, 44.91% and 49.64%, respectively, did not have wives. It should be further noted that among those that reported STIs, 49.12% had one wife, while 5.61% had two wives. In the combined data, 32.74% were not absent from home within the one year prior to the survey. Also, among those that reported STIs, 43.16% was away from home for one to four times, as against 40.17% for those that did not report infection. The results further showed that among men that reported STIs, 70.53% was involved in extra-marital affairs as against 50.36% among those that did not report infections. In the combined data, 42.53% of the men was involved in extra-marital affairs.

**Table 2:** Home absenteeism and men’s number of casual sex partners.

Variables	Had No STIs			Had STIs		All Respondents	
	Frequency		%	Frequency	%	Frequency	%
Number of wives							
0.00	3428		49.64	128	44.91	3556	49.45
1.00	2968		42.98	140	49.12	3108	43.22
2.00	416		6.02	16	5.61	432	6.01
3.00	79		1.14	1	0.35	80	1.11
4.00	11		0.16	0	0.00	11	0.15
5.00	4		0.06	0	0.00	4	0.06
Number of times away from home							
None	2304		33.36	50	17.54	2354	32.74
1 < 5	2774		40.17	123	43.16	2897	40.29
5 < 10	768		11.12	41	14.39	809	11.25
10 < 15	420		6.08	29	10.18	449	6.24
15 < 20	137		1.98	10	3.51	147	2.04
20 < 25	195		2.82	12	4.21	207	2.88
25 < 30	33		0.48	0	0.00	33	0.46
30 < 35	49		0.71	5	1.75	54	0.75
35 and above	226		3.27	15	5.26	241	3.35
Number of sex partners excluding spouse in past one month							
None	4049	58.63		84	29.47	4133	57.47
1 < 5	2689	38.94		166	58.25	2855	39.70
5 < 10	127	1.84		24	8.42	151	2.10
10 < 15	19	0.28		3	1.05	22	0.31
15 < 20	10	0.14		2	0.70	12	0.17
20 < 25	3	0.04		5	1.75	8	0.11
25 < 30	3	0.04		0	0.00	3	0.04
30 < 35	2	0.03		1	0.35	3	0.04
35 and above	4	0.06		0	0.00	4	0.06

Table 3 presents the proportions of men that were aware of STIs and their level of knowledge about transmission and preventive methods. It reveals that majority of the respondents were aware of STIs and AIDS. In order to reduce the risk of contracting HIV, 75.58% and 84.58% of all the respondents indicated that condom should be used during sex as well as having one sex partner. However, wrong impressions of contracting HIV through mosquito bites and through sharing of food with infected persons were reported by 31.94% and 12.44% of the respondents, respectively. A majority of the respondents (83.49%) noted that healthy looking persons could have HIV. Among those that reported STIs, 33.33%, 30.18% and 13.33%, respectively, used condom during sex with their most recent partner, second to most recent partner and third to most recent partner as compared to 24.69%, 15.04% and 4.17% among those that did not report STIs. 

**Table 3:** Knowledge of STIs among the men and use of condom.

Variable	No STI reported	STI reported	All
Ever heard of a Sexually Transmitted Infection (STI)	98.70	100.00	98.75
Ever heard of AIDS	98.29	99.65	98.35
Reduce risk of getting HIV: always use condoms during sex	75.34	81.40	75.58
Reduce risk of getting HIV: have 1 sex partner only, who has no other partners	84.69	81.75	84.58
Can get HIV from mosquito bites	31.61	40.00	31.94
Can get HIV by sharing food with person who has AIDS	12.90	11.23	12.44
A healthy looking person can have HIV	83.28	88.77	83.49
Condom used during last sex with most recent partner	25.69	33.33	25.03
Condom used during last sex with second to most recent partner	15.04	30.18	15.64
Condom used during last sex with 3rd to most recent partner	4.17	13.33	4.53
Number of sex partners, excluding spouse, in last 12 months	0.76	2.14	0.82

Table 4 presents the logistic regression results. It reveals that the model produced a good fit for the data given the statistical significance of the likelihood ratio (LR) chi-square (*p* < 0.01). This also indicates that the parameters of included variables were not jointly equal to zero. In the standard logit results, the parameter of away from home for more than one month variable showed statistical significance (*p* < 0.01). The odd ratio of this parameter implies that those that were away from home for more than one month had risk of being infected with STIs significantly higher by 1.449 (*p* < 0.05) as compared to those that were not away from home. The result further showed that the parameter of number of living children has a negative sign. This implies that if the number of living children increases by one person, the odd of being infected with STI increased significantly by 0.9284 (*p* < 0.05). 

**Table 4:** Logistic Regression Results of Factors Influencing Morbidity with STIs.

Variables	Standard Logit Parameters		Odd-Ratio	
	Parameter	*t*-value	Parameter	*t*-value
Urban residence	−0.0848	−0.62	0.9187	−0.62
Years of education	0.0068	0.49	1.0068	0.49
Gender of household head	−0.2602	−1.41	0.7709	−1.41
Age of household head	−0.0063	−1.35	0.9937	−1.35
Read newspapers	0.1281	0.94	1.1367	0.94
Listening to radio	−0.1566	−0.9	0.8550	−0.9
Watching TV	0.3730	1.67	1.4521	1.67
Times away from home	0.0052	1.31	1.0052	1.31
Away for more than one month	0.3709	2.68	1.4490	2.68
Partner pregnant	−0.0760	−0.33	0.9269	−0.33
Number of living children	−0.0743	−2.31	0.9284	−2.31
Knowledge of any contraceptive	1.1496	1.13	3.1570	1.13
Smoke nothing	−0.3670	−2.44	0.6928	−2.44
Circumcised	0.9552	1.86	2.5991	1.86
Married	0.9021	4.32	2.4647	4.32
Use condom to prevent HIV	0.1434	0.86	1.1542	0.86
Have one sexual partner	−0.4162	−2.47	0.6595	−2.47
Mosquito bites give HIV	0.3730	2.84	1.4521	2.84
Sharing of food gives HIV	−0.2389	−1.17	0.7875	−1.17
Healthy person can have HIV	0.2412	1.19	1.2728	1.19
Condom use with second most recent sexual partner	0.1926	1.32	1.2124	1.32
Condom use with third most recent sexual partner	0.6101	4.21	1.8406	4.21
Number of sexual partners	−0.1966	−1.19	0.8215	−1.19
Constant	−5.3909	−4.65	0.0046	−4.65
LR chi^2^(24) = 150.52				
Prob > chi^2^= 0.0000				
Pseudo *R*^2^ = 0.0628				
Log likelihood = −1124.0225				

Also, those men without smoking habits had significantly lower odds of being infected with STIs (0.6928, *p* < 0.10) when compared with those that were smoking. However, the odds of being infected with STIs significantly increased (*p* < 0.10) by 2.599 among those men that were circumcised when compared to those that were not. Also, being married significantly increased the odds of being infected with STIs by 2.4647 (*p* < 0.01) when compared with those that were not married. Out of the variables that were included to capture knowledge of STIs and HIV, only two showed statistical significance (*p* < 0.05) thereby informing rejection of the stated null hypothesis. The odds ratio parameter implies that compared to those with many sexual partners, the odds of being infected with STIs significantly increased by 0.6595 (*p* < 0.05) among the men that had one sexual partner. However, those that said that mosquito bites cause HIV have significantly higher odds of STIs by 1.4521 (*p* < 0.01). One of the two variables that captured condom use showed statistical significance with positive sign (*p* < 0.01). The results showed that those men that used condoms with the third most recent sexual partners had significantly higher (by 1.8406) odds of being infected with STIs.

## 4. Discussion

The proportion of the interviewed men that reported STIs within the year prior to the survey was 3.96%. This is also close to the adult HIV prevalence rate of 4.5% estimated for Cameroon in 2012 [24]. Low prevalence of STIs as reported by the respondents could be a reflection of high level of reported awareness [25,26]. Although the infection rate was quite small, the result should be taken with some caution. This is because several issues can affect reliability of data on STI infections. Specifically, there are some asymptomatic STIs whose presence will only be detected through voluntary laboratory testing since they do not have noticeable signs of infection [27]. Second is the fact that not many people would go for such testing due to reality of the aftermath social stigmatization [28].

Understanding the socio-economic characteristics, nature of sexual behavior and knowledge of HIV of the men is critical for designing interventions and programmes that would address the problem. From the results, more male headed households did not report STIs. Depending on the family structure, presence of a man as the head of the household could instill more sexual discipline in the adolescents within that family [29]. It had been emphasized that among adolescents, parental influences constitute a strong motivation for displayed sexual behavior and attitudes [30,31].

Formal education can influence STIs through safeguarding risky sexual behavior [32,33]. In the results, however, men that reported STIs had higher average years of formal education. This is contrary to the findings from a study on HIV prevalence in India [34,35]. It also raises some concerns because it is expected that educated people would have proper knowledge of how to be protected from STIs. Similarly, access to information through radio, newspaper and television was also higher among the men that reported STIs. This obviously raises some concerns and points at the need to ensure that knowledge translates into good actions and attitudes. 

Furthermore, in the combined data, average age of household heads was 44.28 years, while that for men with STI infection was 42.53 years. The results did not show any statistical significant difference in the ages of the men that reported STIs and those that did not. Age is an important factor in STIs because among adolescent people (16 and 24 years of age), once sexual activities commence, their sexual health becomes poorer with higher probability of engaging in unprotected sex [27,28]. However, depending on some cultural norms, individual preferences and level of marital infidelity, older people are likely to have many wives although their sense of perceiving risky sexual activities may be higher. However, this is not vividly reflected in the findings that indicated that married men had significantly higher probability of reporting STIs. It is also important to note that men that reported STIs had slightly higher average number of wives when compared to those that did not report infections. 

The results further showed that although there was good knowledge of HIV transmission among the men, some misconceptions still existed. Proper knowledge of having one sex partner to prevent HIV significantly reduced STI infection. This goes in line with *a priori* expectations because if knowledge translates into actual behavior, understanding the need to stick to one partner is sufficient to enhance fidelity in a marriage relationship, which is obviously a guard against STIs [25,27]. Possession of wrong knowledge that HIV is transmitted through mosquito bites also increased the probability of contracting STIs significantly. However, in the logistic regression results, irrespective of whether condoms was used or not, the results showed a higher likelihood of the men that were involved in multiple sexual relationships to report STIs.

Absenteeism from home could be an important factor for predicting STIs if partners are not able to control their sexual urges while away from home. The results showed that men that were infected with STIs had been away from home more times than those that did not report infection. Smoking was reported by a higher proportion of the men that reported STIs. Similarly, non-indulgence in smoking significantly reduced the probability of being infected with STIs. The premise for understanding the relationship between smoking and STIs can be considered from the viewpoint of likelihood of smoking habit to be associated with drinking alcohol. In some instances, smoking is associated with visitation to prostitutes and other public places where sex can be purchased whether safe or not. The results showed that a higher proportion of the men that reported STIs was circumcised. There are different notions in literature on the linkage between circumcision and STIs. 

## 5. Conclusions

Several initiatives to address growing problem of sexually transmitted infections in Cameroon have been recently undertaken. The slow pace of success is however of significant concern given the socio-economic burdens associated with many of these diseases at the household and national economy levels. In this study, incidence of STIs had been studied with focus on the impact of some sex-related behaviours and HIV knowledge. The findings of the study have pointed at the need for ensuring better integration of issue related to sex education in school curriculums in Cameroon. Present efforts need to be reevaluated and persistently realigned in tune with socio-economic changes and recurring public health challenges in the country. Also, integration of media programmes in a manner that enhances people’s knowledge of risky sex-related behaviors and need for fidelity would go a long way in paving directions for destabilizing the grips of infectious sex-related diseases in Cameroon. In addition, programmes that can enhance positive attitudes on issues related to behavior change in relation to the number of sex partners would go a long way in reducing STIs. Such efforts can target non-governmental organizations (NGOs), faith-based organizations, interest groups at the market and job places for effective impact. Although existing laws in relation to smoking exist in Cameroon, weaknesses in relation to their implementation are critical issues of concern. The government should therefore tighten up existing weaknesses in enforcing such laws and similarly engage relevant institutions to intensify campaigns against smoking. This can significantly reduce consumption of alcohol which obviously portends a direct route for involvementin unprotected sex.
